# On the Epidemic Measles of 1808

**Published:** 1809-05

**Authors:** 


### *On the Epidemic Measles of* 1808 ; *by Dr.* Ferguson.

To the Editors of the Medical and Physical Journal.

GENTLEMEN,

In the spring and summer of 1808, the measles were epidemic throughout the whole of England and Scotland, affecting persons of all ages who had never had them; and it would appear from the various bills of mortality, that the number of deaths occasioned have been greater than what we have witnessed for a long period. Not seeing any observations upon this epidemic in your useful Journal, excepting those in your monthly reports, which have been very brief, I have taken the liberty of sending you a few observations that occurred in my practice.

It was the beginning of summer before many cases appeared in Aberdeen, but in the months of June and July there were few families who did not feel their epidemic influence. The measles have been considered by authors to bear much resemblance to the small-pox, and I am of opinion they bear a striking resemblance in many of their phenomena. The accession of both diseases is much alike, and the fever of both is considered of the inflammatory kind. From what I have observed in the cases I attended, like the small-pox, they may also be distinguished into the mild and confluent. This is most evident when they rage as an epidemic. In some cases I have seen them mild, and others of the most confluent nature, and, like the small-pox, requiring a different treatment for each kind. In the mild sort children got better without much attention or medicine, but in the confluent kind the greatest care was necessary; it was this kind that generally proved fatal. This disease is mentioned by authors, as being of an inflammatory nature. The mild kind of measles, like the distinct small-pox, certainly has the appearance of an inflammatory nature, and requires a treatment as such; but in the confluent measles and small-pox, symptoms of debility so quickly succeed the period of accession (or what has been considered as synocha), that treatment purely antiphlogistic can scarcely be admitted. This disease is considered as occurring principally in the spring, and disappearing in summer. The spring has been cold in Aberdeen for many years, and the spring of 1808 was peculiarly so; but the measles were more violent and epidemic during the warm months of summer, and, I believe, very few cases appeared in the cold spring months, although it is in the cold spring months that inflammatory complaints generally prevail here. The cold of winter seems to have had the effect of stopping their progress, both in the town and country. In the latter, the measles were more mild, and not so fatal as in the town, which could only be attributed to the patient's enjoying a purer and more salubrious air, and being less under the influence of virulent contagion. The warmth of the weather, and the number liable to be affected, had much influence in making the disease more epidemic. There were a number of people in Aberdeen possessed of the idea, that the measles required great warmth; and although this disease was raging while the weather was uncommonly warm, the thermometer being between 70 and 80, it was not uncommon to meet with the patient in a close room, near a strong fire, and covered with a load of blankets. This could not fail of aggravating all the symptoms; indeed, it might have been obvious to any rational observer, who had no medical knowledge, that keeping patients in a close room, without the admission of a free and pure air, must have been attended with very bad effects. It were to be wished, that instead of the foolish and ridiculous stuff inserted in our daily prints, a few plain directions were given to parents how to manage, and avoid doing what is improper, when an epidemic of this kind rages; by this means many lives might be preserved. In this place I cannot forbear mentioning, that it was no uncommon practice among the poorer sort, when the small-pox were epidemic, to give those affected with them them, warm punch to drink, "to cause the eruption to come out." This pernicious practice must have been attended with the worst consequences, when the small-pox were of an inflammatory nature. The same destructive method was adopted in the measles, to bring out the eruption, and in many cases must have been attended with very bad effects; those who called for medical assistance, no doubt escaped this dangerous practice, which could not be sufficiently discouraged. Popular prejudices are sometimes very difficult to remove; an example of this we have in the vacciniola. How prejudiced are some against that invaluable discovery, which has saved the lives of thousands! And were an antidote discovered for the measles, it would meet with the same opposition.

Authors observe, that the eruption of the measles appears about the fourth day. This holds true with respect to the mild kind, but the eruption in the confluent is oftentimes longer in making its appearance, which is the reverse in the confluent kind of small-pox, where the eruption appears rather more early than it does in the distinct kind. It is very usual too for the eruption of the confluent measles to appear first in greater quantity upon the legs, arms, and face, than upon the breast, back, or belly: I observed that the same often happened in many cases of confluent smallpox. In the measles the danger does not so much depend upon the confluence or quantity of eruption, as upon the violence of the fever; and I am apt to believe, that it was the violence of a certain species of fever which so often rendered the small-pox dangerous and confluent. This is the reason why the vaccine pock is attended with so little danger, it being uncombined or unproductive of much fever. I once saw a case of small-pox where the eruption appeared like flea bites, of a purple colour, which, as they became pustular, were filled with blood, and resembled in colour the ecchymosis from a bruise. From the beginning, the fever in this case was of the putrid kind, and the patient died before the secondary fever came on.

When I was attending the hospital at Aberdeen, there was a man in it very bad with the small-pox; he had many pustules, and after they had begun to blacken, there were several large abscesses which suppurated and burst upon his back and thighs. His body had a very putrid smell. While I was looking at the abscess upon his thigh, I was instantaneously seized with a tremor and sensation of coldness, with slight vertigo. I was obliged to go immediately home; after being there an hour, I felt one of my tonsils tonsils sore and stiff, with some difficulty in swallowing my saliva; next day my throat was very sore; and as I was a pupil of Dr. Traver's at the time, he attended me. It turned out to be a very violent scarlatina anginosa. I was not visiting any person with scarlatina, neither was there any case of this disease in the hospital; but I was as certain of the instantaneous reception of the infection from this case, as if I had been struck with a musket bullet

The first of these cases shows, that small-pox is not always attended with the same kind of fever, and that it is the particular nature of the fever which occasions confluence or danger. The second case evinces, that from the putrefactive process of small-pox, contagion may be produced that will occasion other infectious diseases. It cannot be doubted that contagious diseases are for the most part attended with a certain species of fever, which is capable of propagating, and which has been always considered as generating the same kind of disease. But from some particular temperament, or condition of the body, it may be also capable of producing a different species of infectious fever.

The inference I draw from these observations, is, that measles and small-pox are not always combined with the same species of fever; that although in the distinct kind of both diseases, the fever that attends is of an inflammatory nature, yet in some particular cases it favours very much of the putrid. I attended a case of measles, where the eruptions upon their appearance were of a purple colour, many of which afterwards became more florid ; a number however of very livid petechiæ still continued present, with great prostration of strength ; black, loose, and fœtid stools; tongue covered with black fur ; putrid gums ; pulse quick and weak ; redness of the adnata, and swelling of the eylids and face, with frequent tickling cough, sickness, and vomiting. In this case the eruptions continued out longer than usual, and many of them were pustular, and evidently contained a fluid. I always observed, that in full grown persons the eruptions were more numerous, quicker in appearing, and longer in going off, than in young subjects ; and that although many full grown persons were very ill, yet the measles were more fatal to the young. A very troublesome symptom attended almost every case of measles, where the patient could speak to complain of it, which was a violent pain of the belly ; and it is not unreasonable to suppose that this symptom accompanied the measles in all subjects. It never appeared until until the eruptions were some time out, and often not till they were subsiding. It was frequently accompanied with diarrhœa and vomiting; when this later symptom attended, it was more dangerous. The diarrhœa was attended with tenesmus and a discharge of mucus; these symptoms continued three or four days, and wasted the patients very much, and were most severe in the confluent kind.

The symptoms mentioned above, must have proceeded from great morbid affection of the stomach and intestines, and this affection might probably be an eruptive inflammation of these viscera. It would have been highly gratifying, to have had an opportunity of inspecting the subjects who died of the most violent species, where there was great internal affection; but I never had an opportunity of so doing.

There is, generally, a very violent tickling cough, with more or less of catarrhal expectoration, attends the measles from their first appearance, which increases with the progress of the eruption, and after this subsides it gradually abates; in some cases the lungs are greatly affected, and pneumonic symptoms very violent.

I have seen some children who had got over the measles, and were seemingly recovering, though slowly, suddenly affected with cough and dyspnœa to such a degree as to prove fatal. But in general I observed, that the cough was less violent after the eruptions had subsided; and, when the lungs were not much affected, the cough and catarrhal discharge gradually disappeared. I think that it was not so much the pneumonic affection, as of the chylopoetic viscera that proved most dangerous. I observed also, that children who were sucking, had the disease less violent, than those who were between twenty months and three years of age. Ignorant people blamed the vaccine inoculation for making the measles more violent; but I can testify, that the measles were less violent in subjects who had been inoculated, than in those who had not.

I considered this disease as in general of an inflammatory nature, and my indications of cure were always founded upon this idea. But, if the measles are combined with other species of fevers, which will only happen when they are violently epidemic in warm weather, this will be quickly perceived; and even here the antiphlogistic plan of treatment, judiciously practised, and not pushed to excess, cannot be attended with any bad consequences. And when symptoms of debility present themselves, themselves, the strength of the patient must be supported by food and medicines adapted to the purpose. That symptoms of debility do present themselves in the measles, is acknowledged by those who consider them an inflammatory affection, and is explained upon the principle of sthenic affection changing into asthenic, from the violence of excitement and quantity of eruption acting as a violent stimulus, and inducing indirect debility. How some contagions operate in producing inflammatory diathesis, while others have an opposite action, as in typhus, tussis convulsiva, and plague, is deserving of consideration, which I shall notice in some future communication.

In treating the measles, I observed that whatever lessened the violent motion or excitement of the system, was particularly serviceable, especially blood-letting, which, though not generally practised here in this complaint, I found very beneficial in all the stages, except where there were petechiæ or great prostration of strength. In the treatment, I had in view the three stages of this disease. The stage of accession—of eruption—and desquamation. In the first stage, all the symptoms denote violent disordered action of the functions, or increased excitement. The pulse and respiration quick; the eyes red; pain in the head and back; great thirst, with a sensation of chilliness, while the skin is burning hot; urine high coloured, and voided in small quantity; anorexia, and sickness; frequent tickling cough, and catarrhal spiting. When these symptoms are moderated by the antiphlogistic plan of cure, the second or eruptive stage is more mild. In the beginning, antimonial emetics are very serviceable for cleansing the stomach, increasing the excretions, and moderating the excitement of the system; gentle laxatives of the saline kind were also very useful; but the principal and most powerful means is blood-letting. This seldom fails of abating the increased action of the system, and is attended with this effect, that the eruption which was only partial becomes more general, and appears in greater quantity. One of my children catched the infection from a young man who had a very confluent kind. She fevered on Sunday, and the eruption began to appear upon Thursday. On Friday she was very ill; I bled her with a large leech, which took away a considerable quantity of blood. On Saturday morning her whole body appeared covered with measles, which I attributed to the bleeding. Saturday evening she was rather easier, and the fever a little abated; violent tickling cough attended and swelling of the eyelids, eyelids, which would have been closed with the dry mucus if they had not been frequently bathed with milk and water. On Sunday the fever was some degrees better, and she began to smile. Monday, greatly better, but violent cough. She continued getting better for nine days; on the tenth she was seized with diarrhœa and vomiting, which weakened her more than the measles, and which continued for eight days; after these symptoms abated, she was sent a few weeks into the country, and soon got well. The eruption in the second stage seems to depend much upon the degree of fever present; and if the excitement of the system be moderated, it appears more freely and critical, yet the symptoms are not presently relieved by the eruption. The cough and catarrhal discharge increase; the eyes are more inflamed, and the eyelids swelled; the sickness increases to vomiting; pain in the belly more severe, accompanied with diarrhœa and tenesmus. Delirium frequently attends this stage.

If blood-letting has not been used in the first stage, it will be highly necessary to have recourse to it in the second. It is of the greatest advantage in all the stages of this disease, if the symptoms indicate violent excitement; and notwithstanding its having been employed in the accession, if the fever be not abated, a repetition will be of advantage in the eruptive stage. As it is not very convenient to bleed children with the lancet, owing to the smallness of their veins, leeches will be very proper, and they can be applied to any part; the ankles are most generally used. To relieve the cough in this stage of the disease, several applications have been recommended by medical men, such as mucilaginous mixtures; syrups joined with oil; infusion of linseed and liquorice root, and other pectoral medicines. The tongue and fauces are in an eruptive and inflamed state in this disease, and whatever is of an acrid nature really gives pain. I have seen children refuse currant jelly, orange juice, &c. because they stimulated the inflamed surface of the mouth and throat; mucilaginous medicines, therefore, are certainly of use in covering the surface of the fauces from the acrid humours that proceed from the inflamed mucus glans of the throat, lungs and head. To young children I generally prescribed calf's foot jelly, which served the double purpose of lubricating the inflamed fauces, and as a nourishing food when the stomach would not admit of any thing else. For older subjects, I gave for the cough the cerum cetæ mixture. By this practice, the cough very soon abated in all the the cases which I attended. In the stage of desquamation, pneumonic symptoms are sometimes very troublesome and dangerous, and this more frequently occurs when the disease has been violent, or the lungs previously weak; ricketty children suffer much in this stage. Blistering is very serviceable in relieving the cough and dyspnœa; and although there be an appearance of debility, yet bleeding in small quantity is attended with advantage. The tincture of digitalis has also very good effects when given in this stage, and is often useful in abating the pain in the belly; opiates are also necessary for this very troublesome symptom; and when diarrhœa and vomiting follow the measles, they are the most powerful agents that can be had recourse to. There is a state of convalescence after the measles, which continues a considerable time, and is very distressing to the patient. It consists in a slow kind of fever, with evening exacerbations. I have found a free and pure air, with a milk diet, most serviceable for children in this state; and exercise in the open air for those advanced in years.

---

*** We are much obliged to Dr. Ferguson for these remarks on an epidemic, which has been more generally fatal during the last year than any records afford, excepting the history furnished by Dr. Watson, which was confined to the Foundling Hospital. Dr. F. we are sure, will not be offended if we object to some part of his theory. That the contagions are accompanied with different kinds of fever, in different seasons, and in different people in the same seasons, cannot be questioned. But in the interesting account Dr. F. gives of his own case, we should suspect that the disease in his throat, and subsequent efflorescence on his skin, was the effect of putrid matter applied by effluvia to that semicuticular and highly sanguiferous part, the throat. In this case he might be said to be inoculated with putrid matter, an event which not uncommonly produces a local affection and general efflorescence. Perhaps Dr. F. may inform us, whether the disease with which he laboured, was communicated to others. If such really was the case, our suggestion must be unfounded.

We shall conclude these remarks by again expressing our satisfaction in receiving this history of an epidemic measles in Aberdeen; and also our hopes, that our readers in the fen countries will follow this example, by favouring us with some account of the intermittents and continued fevers, which, from the last summer and autumn, have been so prevalent among them. EDIT.

